# Combination of Agility and Plyometric Training Provides Similar Training Benefits as Combined Balance and Plyometric Training in Young Soccer Players

**DOI:** 10.3389/fphys.2018.01611

**Published:** 2018-11-13

**Authors:** Issam Makhlouf, Anis Chaouachi, Mehdi Chaouachi, Aymen Ben Othman, Urs Granacher, David G. Behm

**Affiliations:** ^1^Tunisian Research Laboratory “Sport Performance Optimization”, National Center of Medicine and Science in Sports (CNMSS), Tunis, Tunisia; ^2^Sports Performance Research Institute New Zealand, AUT University, Auckland, New Zealand; ^3^Division of Training and Movement Science, University of Potsdam, Potsdam, Germany; ^4^School of Human Kinetics and Recreation, Memorial University of Newfoundland, St. John’s, NL, Canada

**Keywords:** jump training, plyometrics, change of direction, adolescents, football

## Abstract

**Introduction:** Studies that combined balance and resistance training induced larger performance improvements compared with single mode training. Agility exercises contain more dynamic and sport-specific movements compared with balance training. Thus, the purpose of this study was to contrast the effects of combined balance and plyometric training with combined agility and plyometric training and an active control on physical fitness in youth.

**Methods:** Fifty-seven male soccer players aged 10–12 years participated in an 8-week training program (2 × week). They were randomly assigned to a balance-plyometric (BPT: *n* = 21), agility-plyometric (APT: *n* = 20) or control group (*n* = 16). Measures included proxies of muscle power [countermovement jump (CMJ), triple-hop-test (THT)], muscle strength [reactive strength index (RSI), maximum voluntary isometric contraction (MVIC) of handgrip, back extensors, knee extensors], agility [4-m × 9-m shuttle run, Illinois change of direction test (ICODT) with and without the ball], balance (Standing Stork, Y-Balance), and speed (10–30 m sprints).

**Results:** Significant time × group interactions were found for CMJ, hand grip MVIC force, ICODT without a ball, agility (4 m × 9 m), standing stork balance, Y-balance, 10 and 30-m sprint. The APT pre- to post-test measures displayed large ES improvements for hand grip MVIC force, ICODT without a ball, agility test, CMJ, standing stork balance test, Y-balance test but only moderate ES improvements with the 10 and 30 m sprints. The BPT group showed small (30 m sprint), moderate (hand grip MVIC, ICODTwithout a ball) and large ES [agility (4 m × 9 m) test, CMJ, standing stork balance test, Y-balance] improvements, respectively.

**Conclusion:** In conclusion, both training groups provided significant improvements in all measures. It is recommended that youth incorporate balance exercises into their training and progress to agility with their strength and power training.

## Introduction

In many court and field sports like basketball, rugby, and soccer, elite versus sub-elite athletes are characterized by better muscle strength and power, balance, linear sprint, and reactive change-of-direction (CoD) performances (Asadi et al., 2017). In previous cross-sectional studies, low-to-moderate correlations were reported between straight sprint tests and various agility and CoD tests with shared variances ranging from 11 to 22% (Mayhew et al., 1989). Moreover, in a systematic review and meta-analysis, Muehlbauer et al. (2015) observed small-sized associations between variables of balance and lower-extremity muscle strength/power, irrespective of the age group considered. Findings from these studies indicate that the examined physical qualities were specific or somewhat independent in nature and should therefore be tested and trained complementarily. Behm et al. (2005) reported significant correlations between stationary balance measures and ice hockey skating speed. The significant correlations in these studies between performance, balance and agility may relate to the deficits in strength, power and movement velocity when unstable (Anderson and Behm, 2005; Drinkwater et al., 2007; Behm et al., 2010a,b). Rapid changes of direction that occur with agility maneuvers, challenge the ability to either maintain or return the centre of gravity over the base of support (metastability) and thus provide a stress to dynamic balance (Kibele et al., 2015). Since the balance of youth is not fully mature and in a stage of maturational development (Payne and Isaacs, 2005), training programs that could enhance static and dynamic balance should enhance athletic performance.

To achieve high performance levels in these physical qualities, training must start at an early age during long-term athlete development (LTAD). LTAD is a structured approach to the training of young athletes with one of the goals to maximize sporting talent by increasing the likelihood of developing a gifted child into a world-class athlete (Lloyd et al., 2015). For this purpose, Granacher et al., 2016 recently introduced a conceptual training model for LTAD to promote physical development of young athletes. Agility training, balance training, and plyometric training constitute major components of this model during different LTAD stages (e.g., FUNdamentals, learning and training to train).

In this context, Chaouachi et al. (2014a) examined whether combined balance and plyometric training compared to single-mode plyometric training produced greater performance improvements on measures of physical fitness in children. Although the combined program only involved half the volume of plyometric training as the single-mode plyometric program, the combined program resulted in better sprint and shuttle run performances as opposed to the single intervention plyometric program. These findings suggest that immature or a lack of optimal balance capabilities might compromise plyometric training adaptations. However, not all training programs that place a stress on stability have proven to be effective. In a time-efficient approach, Granacher and colleagues examined the effects of combined balance and plyometric training in the form of plyometric training on unstable surfaces on measures of physical fitness in pre-pubertal and pubertal young soccer players (Granacher et al., 2015; Negra et al., 2017b). Interestingly, these authors could not find any additional performance enhancing effects following plyometric training on unstable as opposed to plyometric training on stable surfaces. The effectiveness of combining balance and plyometric training programs is further conflicted by a pair of studies by Chaouachi and colleagues who investigated sequencing effects of balance and plyometric training on physical fitness in young soccer athletes (Hammami et al., 2016b; Chaouachi et al., 2017). While the within session sequence of plyometric and balance exercises (i.e., alternated vs. blocked balance and plyometric exercises) did not affect training adaptations (Chaouachi et al., 2017), a clear effect was reported if balance and plyometric training were sequenced on the level of a mesocycle (4 weeks of balance followed by 4 weeks of plyometric training or vice versa). Blocked balance training performed prior to blocked plyometric training (Hammami et al., 2016b) resulted in greater physical fitness improvements compared to blocked plyometric training conducted prior to balance training.

In field sports like soccer, agility represents an important performance determinant that should be included in the training regime of young soccer players (Davids et al., 2000). In a recent systematic review and meta-analysis, Asadi et al. (2017) examined the influence of maturation on agility performance gains after plyometric training. Findings from 16 studies were statistically aggregated and revealed largest effect sizes (ES) for the groups aged 16–18 years (ES = 0.99) and 13–15.9 years (ES = 0.95) compared to the youngest group aged 10–12.9 years (ES = 0.68). Following up on the recent studies of Asadi et al. (2017) and Chaouachi and colleagues (Hammami et al., 2016b; Chaouachi et al., 2017), it can be postulated that the inclusion of agility in young athletes’ training program may result in additional performance enhancing effects as opposed to the combination of static balance and plyometric training.

Agility proficiency would necessitate good dynamic balance. The balance system would need to compensate and adjust as the rapid CoD could repeatedly shift the center of gravity outside the base of support and challenge the equilibrium or metastability (Kibele et al., 2015). This hypothesis is based on the principle of training specificity (Behm and Sale, 1993) which implies that training must attempt to closely mimic the demands of the respective sport specific activity (Behm and Sale, 1993). Two recent studies have shown training-related improvements when combining plyometric training with sprint and change of direction with 14–15 years old (Hammami et al., 2018) and 11–12 year old children (Michailidis et al., 2018). The older youth experienced training improvements with 6 of 8 tests, whereas the 12 years old youth only experienced training advantages with 2 of 8 tests. Perhaps, the more immature balance of the younger children, could not take full advantage of the rapid changes in dynamic balance with agility or CoD training when preceding plyometric training. A less mature balance system may react more positively by beginning with the less challenging static balance progression.

Therefore, the purpose of this study was to examine the effects of combined balance and plyometric training versus combined agility and plyometric training and a control on components of physical fitness in 11–12 years old adolescent soccer players. With reference to the relevant literature (Hammami et al., 2016b; Chaouachi et al., 2017), we expected that combined agility and plyometric training would result in larger performance improvements compared with combined balance and plyometric training and control because agility compared with balance exercises are more challenging to align the center of mass (CoM) over the base of support.

## Materials and Methods

### Participants

Fifty-seven male elite young soccer players belonging to the same premier league Tunisian soccer club youth academy (Esperance Club Tunis, Tunisia; Champion of the national championship U12 and qualifiers for the world final of Danone Nations Cup), were involved in this longitudinal study. They were randomly assigned to Balance-Plyometric (BPT: *n* = 21), Agility-Plyometric (APT: *n* = 20) training program or a control group (*n* = 16). Only players who participated in at least 85% of all training sessions were included in the statistical analyses. With reference to the studies of Hammami et al. (2016a; 2016b), an *a priori* power analysis (Faul et al., 2007) with an assumed Type I error of 0.01 and a type II error rate of 0.10 (90% statistical power) was conducted for results in the standing stork test as a proxy of balance and revealed that overall 57 participants would be sufficient to observe a medium group × test interaction effect. The physical characteristics and peak height velocity (PHV) stages of all three groups are presented in Table 1. All participants were from similar socio-economic status and had the same daily school and soccer team-training schedules. They all had been playing soccer on a regular basis three-four times a week (i.e., ∼90 min per session) on synthetic surface with a match played during the weekend for more than 3 years.

**Table 1:** Participant anthropometric and peak-height-velocity (PHV, period of maximum rate of growth) characteristics.

	Group APT	Group BPT	Control group
**Age**	11.29 (0.85)	11.06 (0.75)	10.98 (0.80)
**Body mass (kg)**	36.62 (8.26)	36.91 (7.73)	37.22 (7.96)
**Height (cm)**	147.87 (7.43)	145.41 (7.13)	145.33 (5.27)
**Sitting height (cm)**	73.37 (4.07)	71.87 (3.80)	71.58 (2.37)
**Leg length (cm)**	78.29 (5.12)	77.81 (6.21)	78.19 (5.73)
**Predicted PHV (years from PHV)**	–2.60 (0.63)	–2.83 (0.58)	–2.88 (0.48)


*Means and standard deviations (SD) provided (SD in brackets).BPT, balance and plyometric training; APT, agility and plyometric training; PHV, peak-height-velocity*.

Since they lived in the same city, environmental conditions for testing and training were similar for all individuals. Participants were not involved in any after-school activities or any formalized strength and conditioning training programs other than their formal soccer training. To estimate the maturity status of participants, a maturity index (i.e., timing of maturation) was calculated (Table 1) (Mirwald et al., 2002). This assessment is a non-invasive and practical method of predicting years from PHV as a measure of maturity offset using height and age as variables (PHV = -7.999994 + [0.0036124 × age × height]). The equation has previously been validated with standard error of estimates reported as 0.57 and 0.59 years, respectively (Mirwald et al., 2002).

Parents or legal representatives and participants provided informed consent after thorough explanation of the objectives and scope of this project, the procedures, risks, and benefits of the study. This study was carried out in accordance with the recommendations of the Ethics Committee of the National Centre of Medicine and Science of Sports of Tunis (CNMSS) with written informed consent from all subjects. All subjects gave written informed consent in accordance with the Declaration of Helsinki. No player had any history of musculoskeletal, neurological, or orthopedic disorder that might impair their ability to execute plyometric, balance, or agility training or to perform strength, power, balance, agility, or sprint tests.

### Experimental Design

Young male soccer players participated in an 8-week training program with two training sessions per week. After the initial baseline testing session, participants were randomly allocated to a program that either alternated a block of balance exercises prior to a block of plyometric exercises (*n* = 21, BPT) or performed a block of plyometric exercises preceded by a block of agility (*n* = 20, APT) within each training session. Pre- and post-training measures included proxies of power, strength, agility, balance, and speed.

### Procedures

All the players were fully accustomed with the testing procedures used in this research (testing protocols) as they routinely performed these tests in the club as part of their scientific follow-up as described previously (Hammami et al., 2016a,b; Makhlouf et al., 2016; Chaouachi et al., 2017). Before the commencement of the study and prior to the initiation of testing, all players completed a 2-week orientation period (three sessions/week) to become familiar with the general environment, form and technique of each fitness test used to evaluate power, strength, agility, sprint, and balance technique for each training exercise, equipment, and the experimental procedures to minimize the learning effect during the course of the study. During this time, the players received consistent instructions from certified strength and conditioning specialists on proper technique for the agility drills, balance exercises, plyometric exercises and landing. Each participant’s standing and sitting height, and body mass were collected. Height and body mass were assessed using a wall-mounted stadiometer (Easy Glide Stadiometer Perspective Enterprises, Portage, Michigan) and an electronic scale (LifeSource Model UC-321P, A&D Company, Tokyo, Japan), respectively. Afterwards, participants’ performances were tested pre- and post- the 8-week training period. Prior to testing, participants completed a warm-up that included a 5-min jog at a self-selected comfortable pace followed by a 5-min series of dynamic stretching (i.e., hip flexion/extension, hip abduction/adduction, butt kicks). Testing was conducted pre- and post-training at the National team club: Esperance Club Tunis, Tunisia. Pre- and post-training measures were randomized and included proxies of muscle power [countermovement jump (CMJ), triple-hop-test (THT) for distance], muscle strength [reactive strength index (RSI), maximum voluntary isometric contraction (MVIC) of back extensors, knee extensors], agility [4-m × 9-m shuttle run, Illinois change of direction test (ICODT) with and without the ball], balance (Standing Stork Test, Y-Balance Test), and speed (10–30 m linear sprints).

Post-testing was performed three to 5 days after the last training session to ensure optimal recovery (Weiss and Coney, 2003), using the same timeline and procedures as during pre-testing. Furthermore, the training intensity was tapered so that fatigue would not be a factor during post-testing (Ebben et al., 2010). Each testing session was conducted over 3 separate days. Testing was completed at the same time on each testing day for both pre- and post-tests, at the same turf pitch, with the same sport clothes, and by the same trained investigators.

### Training Program Interventions

After pre-testing, participants were randomly assigned to one of 3 groups. BPT participated in an 8-week combined balance and plyometric training program that was performed twice per week (16 training sessions in total). The single training session was conducted in the form of blocked training sessions (block of all balance exercises before plyometric training exercises) as previously described by Chaouachi et al. (2017). The APT group performed a block of agility exercises prior to a block of plyometric exercises within each training session while the control group conducted approximately the same volume of regular soccer training. Groups were matched for age, maturation status, and physical characteristics. To ensure equal training volume between experimental groups, the volume (training weeks, sets, repetitions, and duration) of work during training was matched between balance and agility training. The plyometric training was similar between groups in terms of volume and intensity. The same dynamic warm-up, involving a low-intensity jog; dynamic stretching; low-intensity forward, sideways, and backward running; several acceleration runs; jumping at a progressively increased intensity; and a range of mobility exercises that provided appropriate activation of the lower-limb musculature (Hammami et al., 2016b; Chaouachi et al., 2017) was used before every session by all subjects.

The balance training program (BTP) consisted of 5 exercises, including: (a) Standing with knee on Swiss ball progressing from eyes opened to eyes closed, (b) unilateral and bilateral standing on an inflated disk progressing to a squat exercise, (c) supine straight leg bridge on a Swiss Ball, (d) lunge performed on a foam surface progressing to a BOSU ball or inflated disk while holding dumbbells, and (e) performing bilateral squat with elastic band straps (Theraband, Akron, OH, United States) attached to a bar placed on the shoulders while standing on a foam surface progressing to a BOSU ball or inflated disk (Table 2A). Each task was 30–40 s and was repeated 1–3 times with 8–12 repetitions with 20-s rest between sets. Each stabilization task was performed with the objective to retain balance. The degree of difficulty was progressively increased according to the progress of the subjects.

**Table 2A:** Balance training program exercises.

Balance training and exercises	Week 1	Week 2	Week 3	Week 4	Week 5	Week 6	Week 7	Week 8
Standing with knee on Swiss ball progressing to closed eyes execution	1 × 30	2 × 30	2 × 40	2 × 45	1 × 30	3 × 40	3 × 45	1 × 30
Unilateral and bilateral standing on inflated disk progressing to squat	1 × 8/Leg	2 × 10/Leg	2 × 12/Leg	2 × 15/Leg	1 × 10/Leg	3 × 12/Leg	3 × 15/Leg	1 × 10/Leg
Supine straight leg bridge on Swiss Ball	1 × 8/Leg	2 × 10/Leg	2 × 12/Leg	2 × 15/Leg	1 × 10/Leg	3 × 12/Leg	3 × 15/Leg	1 × 10/Leg
Lunge on foam surface progressing to BOSU ball or inflated disk with holding dumbbells	1 × 8/Leg	2 × 10/Leg	2 × 12/Leg	2 × 15/Leg	1 × 10/Leg	3 × 12/Leg	3 × 15/Leg	1 × 10/Leg
Bilateral squat with elastic straps attached to bar placed on shoulder on foam surface progressing to BOSU ball or inflated disk	1 × 8	2 × 10	2 × 12	2 × 15	1 × 10	3 × 12	3 × 15	1 × 10


Plyometric training was performed with 1–3 sets of 8–15 repetitions according to the recommendation and the training guidelines for pediatric population (Behm et al., 2008; Lloyd et al., 2011), as previously described (Hammami et al., 2016b; Chaouachi et al., 2017). Plyometric drills included standing vertical and horizontal jumps, lateral jumps, ankle hops, skipping, single-leg hopping, maximal hopping, and low-level drop jumps (20 cm) (Table 2B). Because of the relative lack of plyometric experience, verbal feedback in the initial stages was focused on correcting takeoff and landing mechanics. Progressive overload principle was incorporated into the program by increasing the number of foot contacts and varying the complexity of the exercises. For all rapid stretch shortening cycle plyometric exercises, subjects were instructed to give maximal efforts with minimal ground contact times.

**Table 2B:** Plyometric training program exercises.

Plyometric exercises	Week 1	Week 2	Week 3	Week 4	Week 5	Week 6	Week 7	Week 8
Countermovement jumps	1 × 8	2 × 10	2 × 12	2 × 15	1 × 8	n/a	n/a	n/a
Drop jumps + 1 step	1 × 8	2 × 10	2 × 12	2 × 15	1 × 10	n/a	n/a	n/a
Horizontal line jumps	1 × 8	2 × 10	2 × 12	2 × 15	1 × 10	n/a	n/a	n/a
Lateral hops	1 × 8	2 × 10	2 × 12	2 × 15	1 × 10	n/a	n/a	n/a
Ankle jumps	1 × 8	2 × 10	2 × 12	2 × 15	1 × 10	3 × 12	3 × 15	1 × 10
Single leg cone jumps front to back and side to side	n/a	n/a	n/a	n/a	n/a	3 × 12/Leg	3 × 15/Leg	1 × 10/Leg
Single leg maximal rebounding hops	n/a	n/a	n/a	n/a	n/a	3 × 12/Leg	3 × 15/Leg	1 × 10/Leg
Hurdle jumps	n/a	n/a	n/a	n/a	n/a	3 × 12	3 × 15	1 × 10
Drop from a low platform and perform ballistic -type push-ups or clapping push-ups	n/a	n/a	n/a	n/a	n/a	3 × 12	3 × 15	1 × 10


Agility training involved a number of preplanned COD and reactive agility movements commonly used by players seeking to improve agility. Directional change drills included ladder drills (forward sprint with high knee flexion, lateral shuffle, hop in and out, quick-feet ladder sprint, one foot out/2 feet in, carioca), cone drills (oblique shuttle runs, agility T drill, forward T drill, and backward T drill) whilst reactive agility drills used change of direction drills in response to an external stimulus (Table 2C).

**Table 2C:** Agility training program exercises.

Agility exercises		Week 1	Week 2	Week 3	Week 4	Week 5	Week 6	Week 7	Week 8
**Ladders drills:**									
**Forward sprint with high knee flexion**	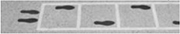	1 × 10s	2 × 10s	3 × 10s	3 × 10s	1 × 10s			
**Lateral high knee flexion:** Lateral shuffle	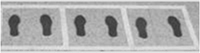	1 × 10s	2 × 10s	3 × 10s	3 × 10s	1 × 10s			
**Hop in and out of steps with hip rotation**	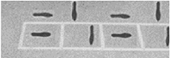	1 × 10s	2 × 10s	2 × 10s	3 × 10s	1 × 10s			
**Forward sprint with high knee flexion:** Quick-feet ladder sprint	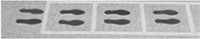						4 × 10s	5 × 10s	1 × 10s
**Lateral high knee flexion:**	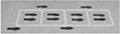						4 × 10s	4 × 10s	1 × 10s
One foot out/2 feet in carioca	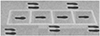						4 × 10s	3 × 15s	1 × 10s
**Oblique shuttle runs:** Forward 5 m backward 5 m, forward 5 m and backward 5 m running Forward 5 m and then crossover strides 5 m and forward 5 m and then crossover strides 5 m	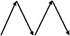	1 × 4	2 × 5	2 × 6	2 × 7	1 × 5	3 × 6	3 × 7	1 × 4
**Agility T drill:** On the start command they run to their right and back 5 m, run forward and back 5 m, run backward and back 5 m and then to the left and back 5 m at full speed	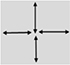	1 × 2	1 × 5	1 × 6	1 × 7	1 × 2	2 × 5	2 × 6	1 × 2
**Forward T drill:** Sprint forward 5 m and move laterally 5 m upon command (verbal or visual, for example coach points in a direction left or right). **Backward T drill:** backward 5 m and move laterally 5 m upon command.	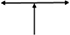	1 × 8	2 × 10	2 × 12	2 × 15	1 × 10	3 × 12	3 × 15	1 × 10
**Square cone drill:** Sprint forward 5 m, shuffle laterally 5 m, sprint backward 5 m and shuffle laterally to starting point.	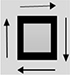	1 × 4	2 × 5	2 × 6	2 × 7	1 × 5	1 × 6	1 × 7	1 × 2
**X Square cone drill:** Sprint forward 5 m, sprint back with crossover strides obliquely to the back right corner 5 m, sprint forward 5 m and sprint back with crossover strides obliquely to the back left original corner 5 m.	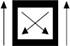


*S × R = series × repetitions with a rest of 1 min*.

The training programs were periodized appropriately by adjusting the technical complexity and intensity of the drills from week-to-week. The first researcher supervised all training sessions, provided coaching and teaching advice, and ensured correct technique was used for each drill. Verbal encouragement was also provided by the researchers during each training session. Tables 2A–C displays the training programs for each of the protocols.

### Physical Fitness Tests

#### Muscular Power

The CMJ was performed using an Ergo jump system (Ergojump apparatus; Globus Italia, Codogne, Italy) according to the procedures described by Chaouachi et al. (2014a; 2014b; 2017). During CMJ, participants were instructed to place their hands on the hips to minimize lateral and horizontal displacement during performance, to prevent any influence of arm movements on the vertical jumps, and to avoid coordination as a confounding variable in the assessment of the leg extensors’ neuro-muscular performance. Participants also had to leave the ground with the knees and ankles extended and land in the same position and location to minimize horizontal displacement and influence on flight time. The CMJ involved the participants lowering themselves as quickly as possible from an upright standing position to a self-selected depth, followed immediately by a vertical jump. Participants were instructed to “jump as high as possible.” Three trials were performed with approximately 2 min. recovery and the best result was used for further analysis.

For the THT, a tape measure was fixed to the ground, perpendicular to a starting line. Participants were instructed to stand behind the starting line with their non-dominant leg forward and the dominant leg off the ground. The leg used to kick a soccer ball was identified as the dominant leg. The subject performed three consecutive maximal hops forward on the same leg to reach the maximal horizontal distance. Arm swing was allowed. The investigator measured the distance hopped from the starting line to the point where the heel hit on the completion of the third and final hop. Both tests were repeated three times, and the maximum distance achieved during the three trials was recorded in centimeters and was used for analysis.

#### Muscle Strength

Reactive strength index was measured during maximal hopping as previously reported (Dalleau et al., 2004; Hammami et al., 2016b). The maximal hopping protocol involved participants performing five repeated bilateral maximal vertical hops in place on the contact mat. Participants were instructed to maximize jump height and minimize ground contact time. The first jump in each trial was discounted, whereas the remaining 4 hops were averaged for RSI analysis as follows: RSI = Jump height (m)/ground contact time (s) (Flanagan and Comyns, 2008). An electronic metronome provided required frequencies by means of an auditory signal.

Maximum voluntary isometric contraction of the knee extensors (dominant leg) were measured using a calibrated hand held dynamometer (Microfet 2; Hogan Health Industries Inc., Draper, UT, United States). Specifics of the test position, stabilization, and dynamometer placement used in this study were chosen according to the instrument manual instructions as previously described (Ben Othman et al., 2017, 2018; Chaouachi et al., 2017). The dynamometer incorporates a load cell and has a digital display. The hand-held dynamometer was placed perpendicular to the anterior aspect of the tibia, just proximal of the medial malleolus for quadriceps testing and against the Achilles tendon for hamstrings testing. For quadriceps testing, participants were seated on the chair of the leg extension machine, positioned so that both feet were off the ground, with hips and knees both flexed at 90°. The lever arm of the leg extension machine was fixed at 100°. The dynamometer was fixed and stabilized by the examiner between the lever arms of the machine and the specific testing placement position on the tested limb segment. The arm of the leg extension machine was fixed with a maximal load to ensure that participants performed an isometric contraction. Participants were instructed to exert an MVIC against the dynamometer for a period of 3–5 s while the dynamometer was held stationary. Three consecutive trials separated by approximately 1-min for both legs and the highest values were recorded for analysis. High hand-held dynamometry reliability measurements in similar pediatric population in our laboratory have been reported elsewhere (Ben Othman et al., 2017, 2018; Chaouachi et al., 2017).

Maximum voluntary isometric contraction back extensor strength was measured in kilograms using a back and leg dynamometer (Takei, Tokyo, Japan). Participants stood on the dynamometer foot stand with feet shoulder-width apart and gripped the handle bar positioned across the thigh. The chain length on the dynamometer was adjusted so that the legs were straight and the hips were flexed at a 30° angle to position the bar at the level of the patella. Participants were then asked to straighten their backs (i.e., stand upright) without bending their knees and lifted the dynamometer chain, with the pulling force applied on the handle, pulling upwards as strongly as possible. Participants completed three trials, the highest score being recorded as the measurement of maximal back strength under isometric conditions. A 30 s rest interval was provided between each trial. Previous test retest reliability scores have been shown to be reliable in a similar athletic pediatric population (Hammami et al., 2016b; Chaouachi et al., 2017).

Maximum voluntary isometric contraction handgrip strength (kg) was measured using a calibrated hand dynamometer (Takei, Tokyo, Japan) as previously described (Ben Othman et al., 2017, 2018). Participants stood comfortably with the arm adducted at approximately 45°. The dynamometer was held freely without support and did not touch the subject’s trunk, with constant extension of the elbow. The grip span of the dynamometer was adjusted to each participant’s hand size so that the proximal inter-phalangeal joints of the four fingers rested on one side of the handgrip and that of the thumb rested on the other side. Participants were required to exert MVIC strength on the dynamometer. Three trials separated by an approximate 1min rest interval for each hand were performed, and the maximum score for each hand was recorded. Excellent MVIC handgrip strength reliability measurements in children in our laboratory have been reported elsewhere (Ben Othman et al., 2017, 2018).

#### Agility

Agility was evaluated with the 4-m × 9-m shuttle run test and the Illinois Change of Direction Speed Test (ICODT) with and without a ball. These tests evaluate the capacity of subjects to quickly change direction. With participants standing behind a starting line, they started the electronic clock by passing through the first timing gate. At the end of the 9-m section, participants were asked to step with one foot beyond a marker while reversing running direction and sprinting back to the start where the same reversal of movement direction was required. After the fourth 9-m section, the subject passed through the second timing gate to stop the electronic clock. The best time of two consecutive trials was recorded for the statistical analysis. At least 2 min of rest was provided between trials. Previous test retest reliability scores have been shown to be reliable in a similar athletic pediatric population (Hammami et al., 2016b).

The ICODT with and without the ball was used to determine participants’ ability to accelerate, decelerate, turn in different directions, and run at different angles, taking into account ball control skill and management. Apart from high overall validity (Hachana et al., 2014), the test could have an advantage in terms of ecological validity as it includes generic cues that closely replicate the majority of movement patterns performed in soccer (Vescovi and McGuigan, 2008). The dimensions and route directions for the ICODT were applied in accordance with established methods (Negra et al., 2017a). The ICODT involves placing 4 cones to indicate a square area that is 10 m long and 5 m wide. In the center of the area, 4 markers were placed 3.3 m apart. The start and finish gates are positioned on the opposite side to indicate the two running points. Each participant had to run as quickly as possible from the start gate, follow a planned route and slalom through the markers without knocking them down or cutting over them. Participants started from a standing position in a prone position with the chin touching the surface of the starting line. Athletes accelerated for 10 m, turned around and returned back to the starting line, and swerved in and out of the 4 markers, and completed the test by running to the finish gate. Participants were instructed not to cut over the markers but to run around them. If a participant failed to follow these instructions, the trial was stopped and attempted again after a 3 min recovery period. The performance outcome was recorded to the nearest 0.01 s using an electronic timing system (Microgate SRL, Bolzano, Italy). The test was repeated in the same manner with the ball, keeping possession of the ball near the foot throughout the testing phase. The best time of two attempts was considered the ICODT score. The difference between the two scores, ICODT with the ball minus ICODT without the ball, represented the time required for ball control techniques and was the third score obtained from the ICODT, named TECHN-INDEX (Hachana et al., 2014; Fiorilli et al., 2017a,b). Participants performed two maximal attempts with each exercise with at least 2 min rest between tests and trials. The faster time was recorded in seconds.

#### Speed

Acceleration and maximal running speed were evaluated using a stationary 10-m sprint, and 30-m maximal speed test. Stationary 10-m sprint involved sprinting 10-m as fast as possible from a stationary standing start position just behind the first timing gate. Start stance was consistent for each participant. Maximal speed 30-m sprint involved sprinting 30-m as fast as possible from a moving start. Players were located 20 cm behind the start line position and were instructed to run as quickly as possible along the 30-m distance. Time was automatically recorded using photocell gates (Brower Timing Systems, Salt Lake City, UT, United States, accuracy of 0.01 s) placed 0.4-m above the ground. Participants performed 2 trials with at least 2 min of rest between trials. The run with the lowest 30-m time was selected for analysis. Previous test retest reliability scores have been shown to be highly reliable in a similar athletic pediatric population (Hammami et al., 2016b; Chaouachi et al., 2017).

#### Balance

Static balance was assessed utilizing the Stork stand balance protocol (Hammami et al., 2016b). To perform the Stork stand test, participants stood with their opposite foot against the inside of the supporting knee and both hands on the hips. On the command, participants raised the heel of their foot from the floor and attempted to maintain their balance as long as possible. The trial ended if the participant either moved his hands from his hips, the ball of the dominant foot moved from its original position, or if the heel touched the floor. This test was carried out on the dominant leg acting as the standing leg. The test was timed (s) using a stopwatch. The recorded score (duration in seconds) was the best of three attempts. Previous test–retest reliability scores for balance measures from our laboratory with a similar pediatric population have been high (Chaouachi et al., 2014a; Hammami et al., 2016b).

Dynamic balance was tested using the lower quarter Y balance test (YBT). Before the test started, participants’ length of the right leg was assessed while in a supine lying position by measuring the distance from the anterior superior iliac spine to the most distal aspect of the medial malleolus. Further, participants practiced six trials per reach direction to get familiarized with the testing procedures. All trials were conducted barefooted. The protocol used for the completion of the YBT is similar to that described previously (Hammami et al., 2016b; Chaouachi et al., 2017) and has been reported to possess high reliability in a similar pediatric population (Hammami et al., 2016b; Chaouachi et al., 2017). Participants stood on the dominant leg, with the most distal aspect of their great toe on the center of the footplate from the YBT Kit. The participants were then asked to push the reach-indicator block with the free limb in the anterior, posterior medial, and posterior lateral directions in relation to the stance foot on the central footplate, while maintaining their single-limb stance. A test trial was classified invalid if the participants (1) did not touch the line with the reach foot while maintaining weight bearing on the stance leg, (2) lifted the stance foot from the footplate center, (3) lost balance at any point during the trial, (4) did not maintain start and return positions for one full second, or (5) touched down the reach foot to gain considerable support. The variables of interest for the study included the maximal reach for each direction. The average maximum normalized reach across the three directions was calculated in order to record a composite score for each subject. YBT measures were normalized by dividing each excursion distance by the participant’s leg length, then multiplying by 100. Thus, normalized values can be viewed as a percentage of excursions distance in relation to the participant’s leg length (Chaouachi et al., 2014a, 2017). The test was demonstrated by a member of the research team prior to the participant completing three practice trials in each direction. Following the completion of the test trials, each participant was given a 1-min rest period and then conducted two test trials in each direction. The examiner manually measured the distance from the center of the grid to the touch point and the results were documented after each reach. A composite score (CS) was calculated and taken as the dependent variable using the following formula: CS = [(maximum anterior reach distance + maximum posteromedial reach distance + maximum posterolateral reach distance)/(leg length × 3)] × 100.

## Statistics

Data are presented as group mean values and standard deviations. After normal distribution was examined using the Shapiro–Wilk-Test, an independent samples *t*-test was calculated to determine significant differences in between-group baseline values. The sequencing effects of agility, balance, and plyometric training on variables of physical fitness were analyzed in a controlled trial. For this purpose, separate 3 (Groups: BPT, APT, Control) × 2 (Time: pre, post) ANOVAs with repeated measures were computed. When Group × Time interactions reached the level of significance, group-specific and Bonferroni corrected *post hoc* tests were calculated to identify the comparisons that were statistically significant. Additionally, effect sizes (ES) were determined from ANOVA output by converting partial eta-squared to Cohen’s *d*. Moreover, within-group ES were computed using the following equation: ES = (mean_post - mean_pre)/SD. ES were used to ascertain if an effect was practically meaningful. According to Cohen (1988), ES can be classified as trivial (0.00 ≤*d* ≤ 0.19) small (0.20 ≤*d* ≤ 0.49), medium (0.50 ≤*d* ≤ 0.79), and large (*d* ≥ 0.80) magnitudes. The significance level was set at *p* < 0.05. Intraclass correlation coefficients were analyzed to assess the intrasession reliability of the researchers and participants (2 trials performed for each measure at pre-test) (Shrout and Fleiss, 1979). All analyses were performed using Statistical Package for Social Sciences (SPSS) version 23.0.

## Results

All subjects received treatment conditions as allocated and completed the study according to the aforementioned methodology. Participants who completed the training program, attended all training sessions and none reported any training or test-related injury. Average attendance at the training sessions over the 8 weeks Balance-Plyometric and Agility-Plyometric training was 89.6 and 88.7%, respectively. No statistically significant between-group differences were found at baseline for all anthropometric and physical fitness measures.

### Reliability

Table 3 illustrates the intraclass correlation coefficients of all measures. Reliability measures (ICC) ranged from 0.794 to 0.985 for all tests.

**Table 3:** Intraclass correlation coefficients for all included measures.

Measures	Intraclass correlation coefficients
Knee extension MVIC	0.982
Handgrip MVIC	0.964
Back extension MVIC	0.882
ICODT without a ball	0.942
ICODT with a ball	0.985
Agility 4 m × 9 m	0.970
Countermovement jump	0.978
Reactive strength index	0.970
Triple hop test	0.976
Standing stork balance test	0.961
Y-balance	0.794
10 m linear sprint	0.979
30 m linear sprint	0.982


*MVIC, maximum voluntary isometric contraction; ICODT, Illinois change of direction test*.

### Main Effects

All measures displayed significant main effect, large magnitude (effect size) improvements for time (post-test > pre-test) (Table 4). Significant main effects for group were evident with the ICODT with and without a ball (trivial ES), THT (trivial ES), standing stork balance test (small ES), and a near significant effect (*p* = 0.058; trivial ES) with the CMJ (Table 4). With each of these measures the control group was significantly different from the BPT and APT groups.

**Table 4:** Main and interaction effects for all outcome measures.

	BPT			APT			Control group			*p* (Cohen’s *d*)	
	Pre	Post		Pre	Post		Pre	Post		Main effect: time	Interaction: group × time
											
Variables	Mean ± *SD*	Mean ± *SD*	Δ (%)	Mean ± *SD*	Mean ± *SD*	Δ (%)	Mean ± *SD*	Mean ± *SD*	Δ (%)		
**Strength, power**												
Hand grip strength [kg]	16.3 ± 5.3	19.6 ± 6.0	20.6	15.6 ± 3.4	18.9 ± 3.8	21.4	14.7 ± 4.0	16.2 ± 3.1	10.1	0.001 (2.53)	0.022 (0.78)
MVIC back extensor [kg]	56.1 ± 12.0	65.0 ± 1.8	15.9	56.4 ± 9.7	63.5 ± 8.9	12.6	53.0 ± 9.3	58.8 ± 8.8	10.8	0.001 (2.51)	0.261 (0.45)
MVIC knee extensor [kg]	189.2 ± 45.6	263.0 ± 4.9	39	210.9 ± 57.0	297.8 ± 41.4	41.2	210.9 ± 56.1	259.8 ± 51.2	23.5	0.001 (2.75)	0.106 (0.59)
CMJ [cm]	21.8 ± 2.8	25.8 ± 2.7	18.4	20.8 ± 3.1	23.6 ± 3.4	13.4	20.4 ± 3.6	22.1 ± 3.4	8.3	0.001 (3.13)	0.002 (1.03)
RSI [m/s]	0.8 ± 0.3	1.1 ± 0.2	35.4	0.8 ± 0.2	1.1 ± 0.3	32.7	0.8 ± 0.3	0.9 ± 0.3	21	0.001 (1.96)	0.316 (0.42)
Triple hop [cm]	508.2 ± 44.2	555.0 ± 0.6	9.2	499.7 ± 32.2	541.6 ± 36.6	8.4	479.9 ± 43.1	517.8 ± 40.9	7.9	0.001 (3.34)	0.584 (0.29)
**Agility**																							
ICODT [s]	18.16 ± 0.8	17.69 ± 0.8	–2.5	18.00 ± 0.6	17.43 ± 0.6	–3.1	18.38 ± 0.6	18.28 ± 1.0	–0.6	0.001 (1.28)	0.075 (0.63)
ICODT with ball [s]	23.12 ± 1.4	22.51 ± 1.2	–2.6	23.06 ± 1.6	22.34 ± 1.3	–3.1	25.22 ± 2.8	24.40 ± 3.5	–3.2	0.002 (0.89)	0.929 (0.11)
Agility 4^∗^9m [s]	10.49 ± 0.5	10.03 ± 0.3	–4.4	10.46 ± 0.3	9.97 ± 0.4	–4.7	10.43 ± 0.4	10.36 ± 0.5	–0.6	0.001 (1.64)	0.007 (0.90)
**Speed**												
10 m sprint [s]	2.18 ± 0.1	2.12 ± 0.1	–3.2	2.18 ± 0.1	2.12 ± 0.1	–2.9	2.21 ± 0.2	2.22 ± 0.2	0.5	0.005 (0.80)	0.044 (0.70)
30 m sprint [s]	5.42 ± 0.3	5.33 ± 0.2	–1.7	5.39 ± 0.3	5.27 ± 0.2	–2.4	5.50 ± 0.3	5.51 ± 0.4	0.2	0.004 (0.81)	0.049 (0.69)
**Balance**												
Standing Stork Test [s]	3.94 ± 1.8	9.68 ± 3.8	145.0	4.38 ± 1.7	13.61 ± 9.2	210.5	3.12 ± 1.0	4.47 ± 1.7	43.5	0.001 (1.96)	0.001 (1.13)
YBT CS [cm]	71.2 ± 7.1	81.7 ± 5.2	14.7	71.0 ± 6.5	78.7 ± 4.9	10.9	71.4 ± 6.9	73.3 ± 7.7	2.7	0.001 (2.56)	0.001 (1.32)


**FIGURE 1:**
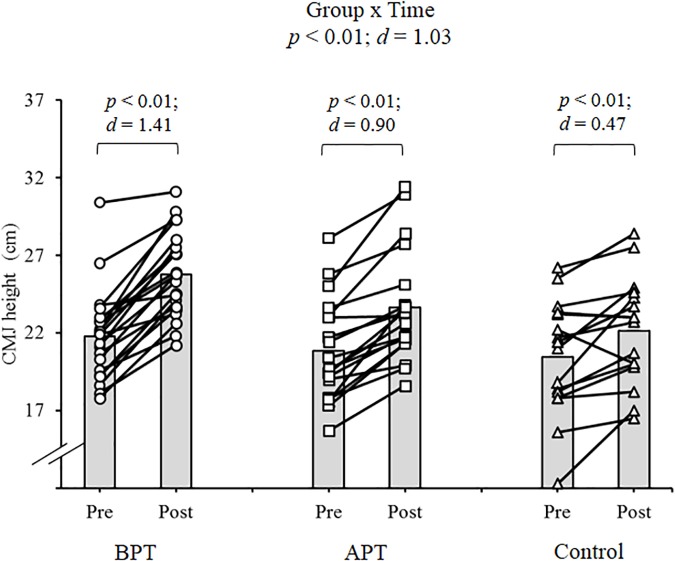
Countermovement (CMJ) height with group means and individual participant scores. BPT, balance and plyometric training; APT, agility and plyometric training.

**FIGURE 2:**
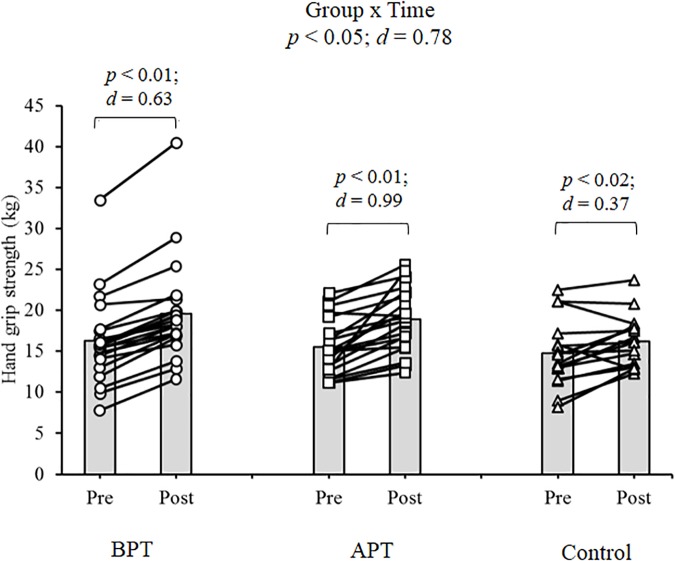
Hand grip maximum voluntary isometric contraction (MVIC) force with group means and individual participant scores. BPT, balance and plyometric training; APT, agility and plyometric training.

**FIGURE 3:**
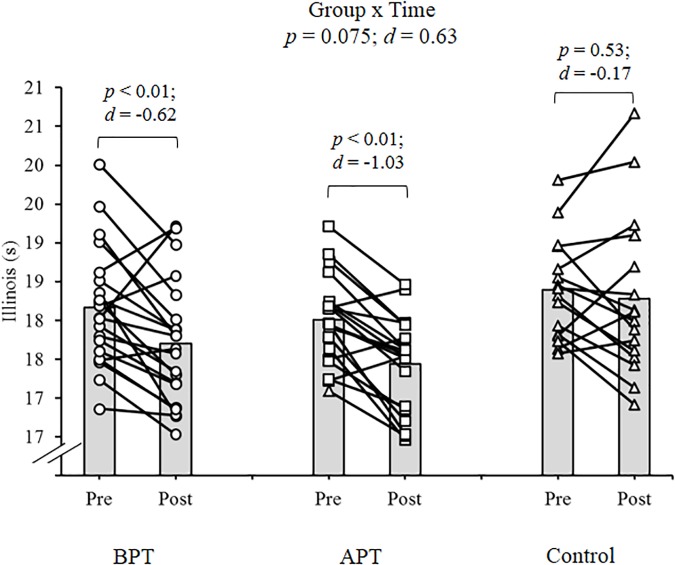
Illinois agility test (ICODT) without a ball with group means and individual participant scores. BPT, balance and plyometric training; APT, agility and plyometric training.

**FIGURE 4:**
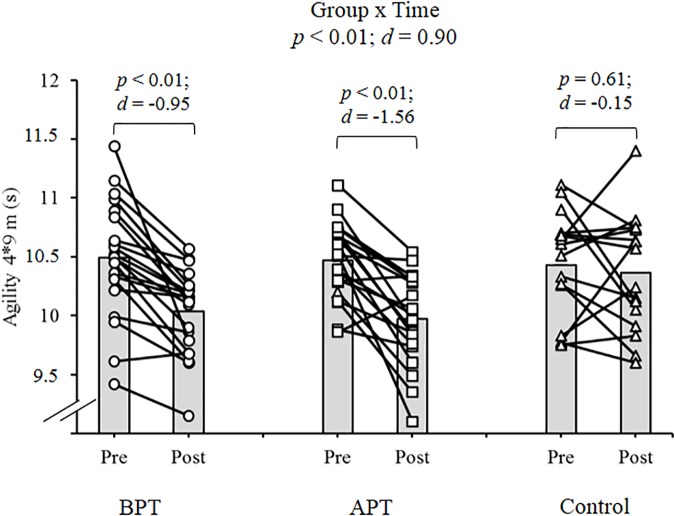
Agility (4 m × 9 m) test with group means and individual participant scores. BPT, balance and plyometric training; APT, agility and plyometric training.

**FIGURE 5:**
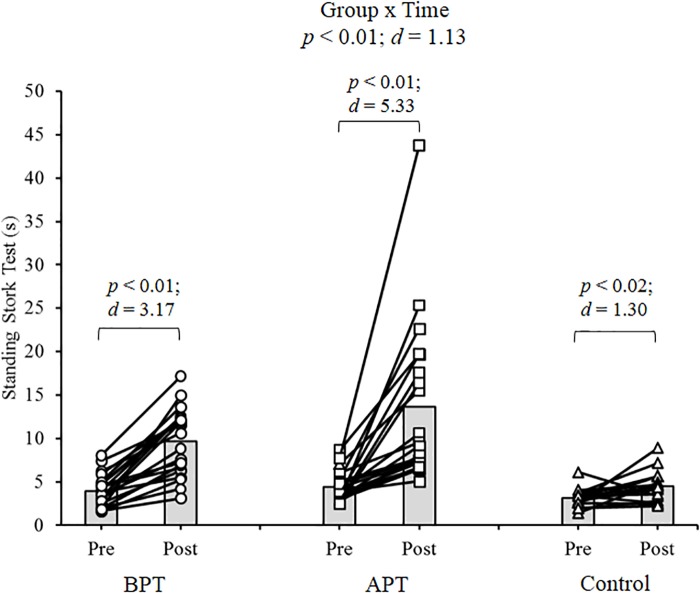
Standing stork balance test with group means and individual participant scores. BPT, balance and plyometric training; APT, agility and plyometric training.

**FIGURE 6:**
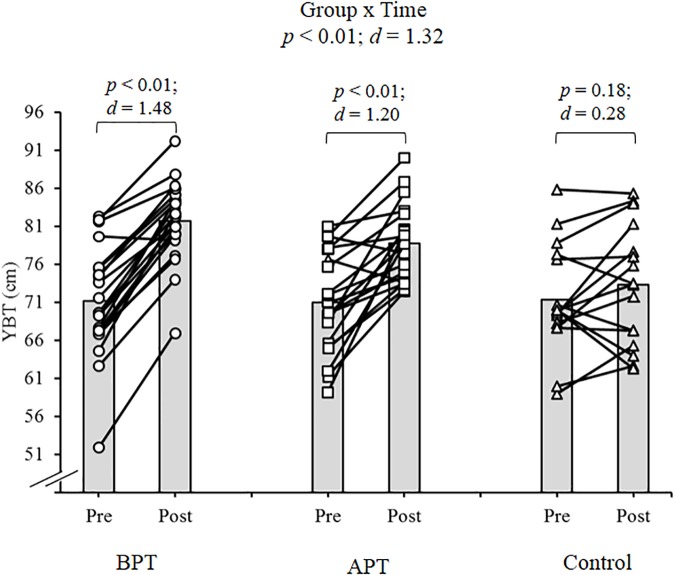
Y-balance test with group composite score (cm) means and individual participant scores. BPT, balance and plyometric training; APT, agility and plyometric training.

**FIGURE 7:**
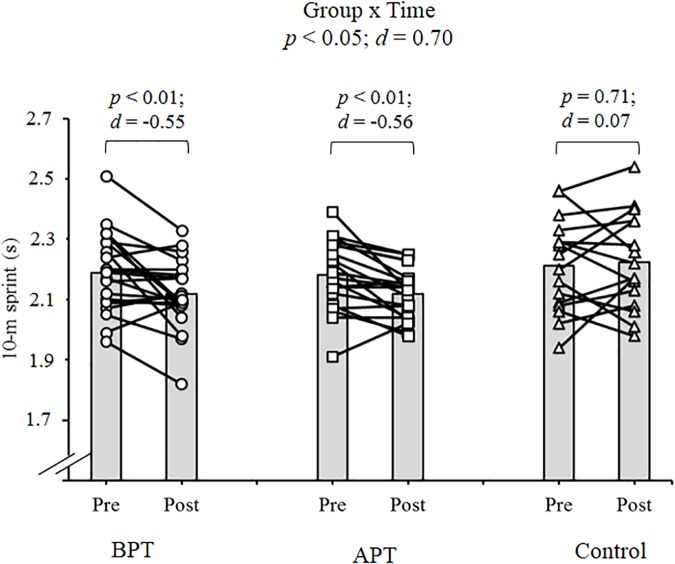
Ten meter sprint time with group means and individual participant scores. BPT, balance and plyometric training; APT, agility and plyometric training.

**FIGURE 8:**
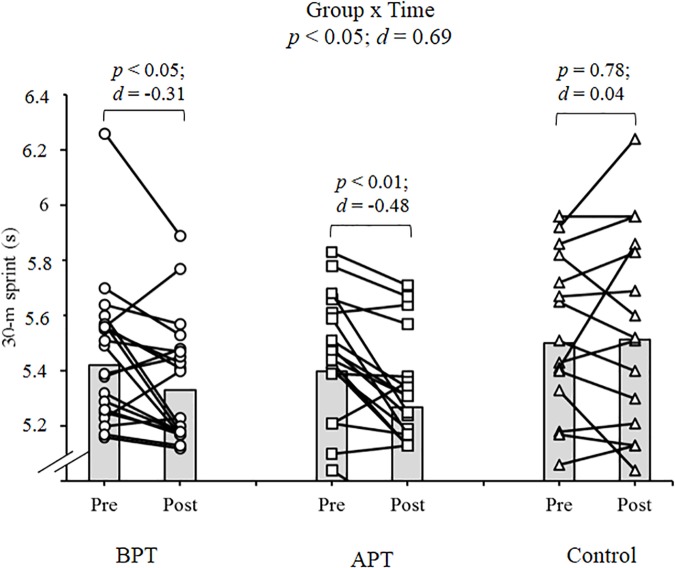
Thirty meter sprint with group means and individual participant scores. BPT, balance and plyometric training; APT, agility and plyometric training.

*The shaded (highlighted) time × group interactions are further illustrated with Figures 1–8 to discriminate the specific significant (or near significant: ICODT) training-related group changes (post hoc analyses).BPT, balance and plyometric training; APT, agility and plyometric training; MVIC, maximum voluntary isometric contraction; YBT CS, Y balance test composite score; ICODT, Illinois change of direction test; CMJ, countermovement jump; RSI, reactive strength index.*

### Interactions

Significant group × time interactions were evident as the APT displayed significant and large magnitude ES pre- to post-test improvements for hand grip MVIC force, ICODT without a ball, agility test, CMJ, standing stork balance test, Y-balance test CS. APT also exhibited significant but only moderate magnitude ES improvements with the 10 and 30 m sprints (Table 4). The BPT group showed small (30 m sprint), moderate (hand grip MVIC, ICODT without a ball) and large magnitude ES [agility (4 m × 9 m) test, CMJ, standing stork balance test, Y-balance CS] significant improvements, respectively (Table 4). Table 4 illustrates all group × time interactions. The figures illustrate the significant (and ICODT near significant) interaction *post hoc* analyses. The *post hoc* analyses show that CMJ (small ES: Figure 1), hand grip MVIC force (small ES: Figure 2), and the standing stork balance test (large ES: Figure 5) improved with the control group. Both training groups experienced significant improvements with ICODT without a ball (Figure 3), agility (4 m × 9 m) test (Figure 4), Y-balance test CS (Figure 6), as well as the 10 (Figure 7) and 30-m sprints (Figure 8).

## Discussion

The most important findings of the present study were the significant and similar BPT, APT improvements following 8 weeks of training versus control for sprinting, change of direction (ICODT), agility, as well as the Y balance test. Optimizing balance and stability is an essential component of sports training. Strength, power, movement velocity and range of motion are adversely affected by an unstable environment (Anderson and Behm, 2005; Drinkwater et al., 2007; Behm et al., 2010a,b). The rationale for the similar training-related strength, power, sprint speed and Y balance improvements relates to the relative immaturity of adolescent balance (Payne and Isaacs, 2005) and thus the inclusion of balance or agility training with plyometrics would enhance these training-related improvements. In the context of balance training, rapid changes in direction (agility and ICODT) training would place stressors on equilibrium or the ability to balance, also leading to balance enhancements. Landing for short periods (<200–300 ms) and then attempting to explode off the surface with sprinting would also challenge an individual’s balance. Hence, the strength and power output when landing on one foot when sprinting, performing the ICODT and 4 m × 9 m agility tests would be impaired with poor balance. The addition of balance or agility training to plyometrics thus enhanced performance in the measures that would be most adversely affected by balance or stability deficits (ICODT, agility, sprints and Y balance test).

The effectiveness of combining balance and plyometric exercises has been demonstrated previously. Hammami et al. (2016b) had 24 young male elite soccer players train twice per week for 8 weeks either with an initial 4 weeks of balance training followed by 4 weeks of plyometric training (BPT) or 4 weeks of plyometric training proceeded by 4 weeks of balance training (PBT). Similar measures were monitored as in the present study and results showed that while both programs improved performance, BPT provided significantly greater improvements with RSI, leg stiffness, THT, and a Y balance test compared to PBT with BPT ES higher with 8 of 13 measures. Another study from this laboratory (Chaouachi et al., 2017) trained 26 male adolescent soccer players over an 8-week training program which alternated individual balance and plyometric exercises or employed a block of balance exercises prior to a block of plyometric exercises within each session. Monitoring similar measures as in the present study, there were significant overall, generally large ES training improvements, but there were insignificant training group differences. Furthermore, McLeod et al. (2009) reported that a 6 weeks neuromuscular training program that included plyometrics and balance exercises improved balance and proprioceptive capabilities of female high school basketball players. Hence, based on the present and prior studies, the inclusion of balance training into youth plyometric training programs is an important component and initial training progression.

A number of youth balance training studies have demonstrated improvements in jumping, running, kicking distance, balance, and agility measures (Behm et al., 2008; Johnson et al., 2011; Granacher et al., 2015). However, according to the concept of training specificity (Behm and Sale, 1993) the greatest transfer effects occur when the training movements most closely resemble the sport activity. The majority of the balance activities in the previously mentioned studies were stationary (Hammami et al., 2016b; Chaouachi et al., 2017). Since soccer and most other sports are dynamic, activities that mimic more closely these demands may provide even better training results.

However, there were no significant differences in the training improvements of the BPT and APT programs in the present study. The similarity of the training adaptations must be related to the balance challenges associated with agility training. The rapid changes of directions often move the individual’s center of mass outside the base of support challenging balance and postural stability. The ability to return the center of gravity within the base of support and maintain balance and postural stability is termed metastability (Kibele et al., 2015) and is a key component of athletic and soccer success. Agility training conceivably also has some advantages over stationary balance training. It is a dynamic, high speed, explosive activity thus adhering to the training specificity concept for soccer. The importance of dynamic balance is further exemplified in a 6-week combined agility and balance training program that reported greater performance enhancements compared to an agility only training group (Zemkova and Hamar, 2010). Hammami et al. (2016a) reported significant correlations of agility with power (standing long jump, CMJ) and sprint (10 and 30 m- sprints) measures as well as balance (Y-balance test). Sekulic et al. (2013) indicated that balance measures were significantly correlated to agility in men but not in women. A number of authors have commented on the balance challenges and benefits of greater balance or metastability associated with change of direction or agility activities (Mirkov et al., 2008; Hammami et al., 2016a). Since high speed change of direction (agility) impose frequent perturbations upon postural control, the ability to efficiently sustain static and dynamic balance (metastability) can positively affect athletic (i.e., soccer) performance (Jones et al., 2009). However, the equivalence of the BPT and APT programs suggests that the physiologically immature balance system of youth (Payne and Isaacs, 2005) in this study adapted to a similar extent independent of the complexity or velocity of the balance challenges. Thus, agility training can be viewed as a plyometric training technique progression that integrates dynamic balance with speed and power.

There were main effects for time for all testing measures indicating general, overall improvements. However, there was a lack of significant interactions for quadriceps and back MVIC, ICODT with a ball, RSI and the triple hop test. Furthermore, the control group only exhibited significant *post hoc* improvements with the CMJ, handgrip MVIC strength and Standing Stork balance test. The three specific performance changes in the control group might be partially attributed to physical contributions from growth and maturation. Furthermore, the prior and ongoing soccer training of these elite players would also play a role. Since soccer involves explosive sprints, jumps and changes of direction (Stolen et al., 2005; Ramirez-Campillo et al., 2014) the legs would already be in a relatively highly trained state (advanced explosive strength) and may have been less susceptible to this short duration training program. Furthermore, since the APT and BPT programs did not involve skill training with a soccer ball, it might be expected that soccer specific coordination skills (i.e., ICODT with a ball) would not be positively affected by additional training. A longer duration or more frequent (3 vs. 2 training sessions per week) training program may be needed to elicit further training adaptations from muscle groups that undergo explosive strength activities on a nearly daily basis. Thus while some improvements must be expected from a sport such as soccer/football that involves agility, change of direction, speed and power, an important point to consider is that adding either agility or balance exercises to plyometric training will provide an even more extensive array of training adaptations.

## Conclusion

In summary, both BPT and APT groups provided statistically significant improvements with sprinting, change of direction (ICODT), agility as well as the Y balance test. Hence, static (BPT) and dynamic (APT) balance training were more effective in the enhancement of measures that may be more adversely affected by instability versus more stable performance measures such as KE, lower back, and handgrip MVICs, and static balance (Stork test). It is recommended based on the present study and prior literature that youth first incorporate balance training exercises into their training regimes whether it be for strength or power and then progress by adding more complex and challenging balance activities such as agility that add greater speeds and torques to the metastable challenges.

## Author Contributions

UG and DB were involved in the conceptualization of the study, data analysis, and the writing of the manuscript. AC and IM were involved in the conceptualization of the study, data assessment, data analysis, and the writing of the manuscript. MC and ABO were involved in the conceptualization of the study, data assessment, and the writing of the manuscript.

## Conflict of Interest Statement

The authors declare that the research was conducted in the absence of any commercial or financial relationships that could be construed as a potential conflict of interest.
